# Interpreting deep learning models for glioma survival classification using visualization and textual explanations

**DOI:** 10.1186/s12911-023-02320-2

**Published:** 2023-10-18

**Authors:** Michael Osadebey, Qinghui Liu, Elies Fuster-Garcia, Kyrre E. Emblem

**Affiliations:** 1 Department of Physics and Computational Radiology, Division of Radiology and Nuclear Medicine, Oslo University Hospital, Sognsvannsveien 20, 0372 Oslo, Norway; 2 Biomedical Data Science Laboratory,Instituto Universitario de Tecnologias de la Informacion Comunicaciones, Universitat Politècnica de València, 46022 Valencia, Spain

**Keywords:** Glioblastoma, Magnetic Resonance Imaging (MR1), 3D Gradient Weighted Class Activation Mapping (3D-Grad-CAM), Deep learning, Convolutional Neural Network (CNN)

## Abstract

**Background:**

Saliency-based algorithms are able to explain the relationship between input image pixels and deep-learning model predictions. However, it may be difficult to assess the clinical value of the most important image features and the model predictions derived from the raw saliency map. This study proposes to enhance the interpretability of saliency-based deep learning model for survival classification of patients with gliomas, by extracting domain knowledge-based information from the raw saliency maps.

**Materials and methods:**

Our study includes presurgical T1-weighted (pre- and post-contrast), T2-weighted and T2-FLAIR MRIs of 147 glioma patients from the BraTs 2020 challenge dataset aligned to the SRI 24 anatomical atlas. Each image exam includes a segmentation mask and the information of overall survival (OS) from time of diagnosis (in days). This dataset was divided into training ($n=118$) and validation ($n=29$) datasets. The extent of surgical resection for all patients was gross total resection. We categorized the data into 42 short (mean $\mu =157$ days), 30 medium ($\mu =369$ days), and 46 long ($\mu =761$ days) survivors. A 3D convolutional neural network (CNN) trained on brain tumour MRI volumes classified all patients based on expected prognosis of either short-term, medium-term, or long-term survival. We extend the popular 2D Gradient-weighted Class Activation Mapping (Grad-CAM), for the generation of saliency map, to 3D and combined it with the anatomical atlas, to extract brain regions, brain volume and probability map that reveal domain knowledge-based information.

**Results:**

For each OS class, a larger tumor volume was associated with a shorter OS. There were 10, 7 and 27 tumor locations in brain regions that uniquely associate with the short-term, medium-term, and long-term survival, respectively. Tumors located in the transverse temporal gyrus, fusiform, and palladium are associated with short, medium and long-term survival, respectively. The visual and textual information displayed during OS prediction highlights tumor location and the contribution of different brain regions to the prediction of OS. This algorithm design feature assists the physician in analyzing and understanding different model prediction stages.

**Conclusions:**

Domain knowledge-based information extracted from the saliency map can enhance the interpretability of deep learning models. Our findings show that tumors overlapping eloquent brain regions are associated with short patient survival.

## Introduction

Gliomas are a group of brain tumors with prognosis and overall survival (OS) that varies with the level of malignancy and patient status. Glioblastoma, one of the most malignant glioma subtypes (isocitrate dehydrogenase (IDH) wildtype), has the worst prognosis where only 5.5 percent of the patients are alive after five years [1, 2]. Therefore, accurate prediction of OS could help physicians develop personalized treatment plans best suited for the individual patient.

Clinical, molecular, and imaging features of glioma are associated with patient prognosis. Clinical factors include age, preoperative Karnofsky performance status, and the extent of surgical tumor resection [3]. Molecular factors include mutations in isocitrate dehydrogenase 1 (IDH1) and O6-methylguanine-DNA-methyltransferase (MGMT) promoter methylation [4]. Magnetic resonance imaging (MRI) is the primary information resource for computer-aided prediction of OS in patients with glioma as it is widely used for diagnosis and follow-up post-treatment examinations [5]. Here, the main regions-of-interest of glioma are primarily the necrosis, edema, and the enhancing tumor. Studies suggest that MRI features can characterize the different subregions of a brain tumor and its association with OS [6]. Imaging features of glioma may therefore provide independent information to complement the clinical variables associated with OS [5].

The recent advent of deep learning to solve large-data regression tasks in healthcare, and particularly in diagnostic imaging, have encouraged several initiatives to predict patient OS by presurgical imaging [7]. Here, a trained model accepts the original MRI data and corresponding ground-truth segmented tissue regions as input features and predicts the OS in a start-to-end manner. Li et al. [8] extracted features at the intermediate layer of the network and combined it with patient age for the final prediction of OS. Suter et al. [9] combined patient age and tumor resection status with image features at the fully connected layer of the network. Han et al. [10] combined features extracted from the output of an attention-based convolutional neural network (CNN) trained for tumor segmentation with patient age to predict OS.

One of the main barriers for the adoption of deep-learning models into the clinical setting are their multiple hidden-layered structure, constituting a ‘black box’ which do not readily represent or contrasts the physician’s prior knowledge of clinically relevant information. Therefore, it is difficult for oncologists to understand how and on what basis the algorithms reach their conclusions. It is important that making deep learning models understandable in the healthcare domain is essential to ensure safety, trust, accountability, ethical compliance, and effective decision-making while mitigating bias and enabling ongoing improvement in healthcare [11–13]. There are, however, several explainable artificial intelligence (XAI) techniques that could contribute to explain how deep-learning models make decisions by incorporating an external unit to monitor the internal mechanism of the model. A popular method, the Gradient-weighted Class Activation Mapping (Grad-CAM) technique, applies a gradient localization map that highlights regions in the input image with significant contribution to model prediction [14]. Another technique called the SmoothGrad averages the gradient sensitivity map of the input data to identify significantly learned features [15]. In the occlusion sensitivity analysis method, portions of the input data are iteratively occluded followed by classification to determine the significant regions of the input data [16]. The Shapley additive explanations (SHAP) gradient explainer combines techniques based on Shapley theory of game theory, integrated gradients, and SmoothGrad into a unified framework [17]. Visualization technique allows the user to visualize convolutional layer channel activations and determine significant features in the input image [18].

Important characteristics of interpretable deep learning models are the visualization of the impact of relevant features and the validation of the prior knowledge they represent [12]. Prior knowledge is very crucial because it allows the physician to establish the link between image features and model prediction. Saliency-based algorithms that express the input image pixels’ contribution to model predictions satisfies only the first requirement. To validate prior knowledge and enhance interpretability, we hypothesize that the saliency maps contain domain knowledge-based information, which, if properly mined, can assist physicians, understand the link between input features and model predictions. Therefore, we derive three Grad-CAM-derived information to enhance its interpretability. The information consist of saliency map, probability of event-of-interest map and brain regions associated with OS classes.

Although current XAI techniques reveal the association between the contributions from pixels in the input data to the model prediction, they suffer from low interpretability because the clinical value of the features extracted by an AI system and its predictions are hard to depict [19, 20]. Visualizations and textual explanations can bridge the gap between user knowledge and the insights provided by XAI algorithms [12, 21]. Therefore, our study proposes to enhance the interpretability of AI-based predictions using a quantitative assessment of the feature spaces in XAI-derived saliency map and anatomical atlas.

## Materials and methods

### Data

**Fig. 1:**
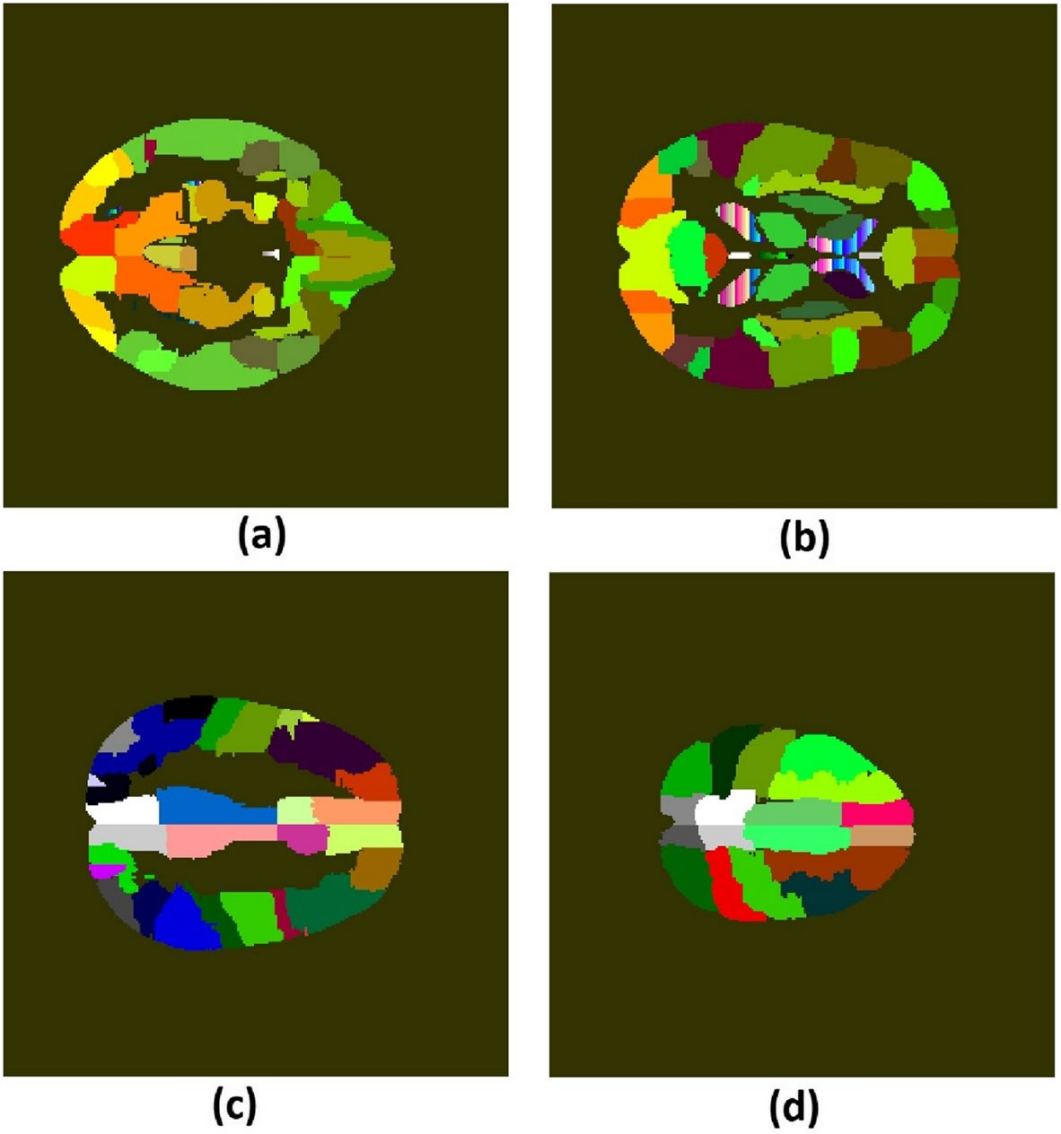
Slice numbers (**a**) 60, (**b**) 80, (**c**) 100 and (**d**) 120 in the SRI 24 brain template showing (**a**) right and left temporal lobes (green) (**b**) lateral ventricle (red-blue-white stripe) (**c**) right precaneus (white) (**d**) right paracentral lobule (white)

Our study includes 118 (training data) and 29 (validation data) preoperative MRI data of subjects with low-grade and high-grade glioma, from the BraTs 2020 challenge dataset [22]. Each training data includes segmentation mask and clinical information on; age, OS, and gross total resection status. Preoperative MRI is suitable for prognosis and diagnosis as it contains de novo information on the location and spatial extent of the tumor within the brain. Each patient data contains four MRI sequences; T1-weighted, T1-weighted post-contrast (T1c), T2-weighted and T2-weighted fluid-attenuated inversion recovery (FLAIR). Each volume data contains 155 slices of in-plane dimensions 240 x 240. The images had undergone standard pre-processing steps, including skull stripping, normalization, bias field correction, and co-registration to the SRI 24 anatomical template [22, 23]. Figure 1a-d shows an example of slices number 60, 80, 100 and 120, in the anatomical template. The average patient age and OS of all patients in this study were 62 years ($\sigma =12$ years) and 14 months ($\sigma =12$ months), respectively. The average OS for the short, medium, and long survivors are 157 ($\sigma =79$), 369 ($\sigma =41$) and 761($\sigma =346$) days, respectively (Table 1).

**Table 1:** Distribution of MRI volumes, average patient age and average overall survival across the three OS classes

Overall Survival Class	Number of MRI Volumes	Range/Confidence Interval Age (Years)	Average Patient Age (Years)	Range/Confidence Interval OS (Days)	Average OS (Days)
**Short-term**	42	29 -87/64- 71	68	12 - 296/132 - 181	157 ($\sigma =79$)
**Medium-term**	30	44 - 86/57- 65	61	300 - 448/354 - 385	369 ($\sigma =41$)
**Long-term**	46	28 - 77/54- 61	57	453 1767/658 - 864	761 ($\sigma =346$)
**Total/Average**	118	28 - 87/60-64	62	12 - 1767/383 - 509	446 ($\sigma =345$)

### Data preprocessing and preparation

We eliminated the 40 and 30 most inferior and superior slices, respectively, in each patient’s MRI volume because they either did not contain any relevant structures or had limited structural information that may compromise the outcome of the features learned by a model. Thus, only 90 of the 155 slices were considered relevant in each patient’s MRI volume. Collectively, the training data from each MRI sequence and its corresponding segmentation mask returned a 3D MRI volume $I_{train}$ containing only tumor regions with dimensions $240 \times 240 \times 90 \times 4$ (image types). The OS for each patient in days was converted to months *T*, which we use to categorize the training data into short-term ($T<10$ months), medium-term ($10< T< 15$ months), and long-term ($T> 15$ months) survivors.

### Training

The two major sections of the 3D CNN are feature extraction, which learns high-level features formed from low-level features, and the classifier, which manipulates the high-level features for classification. The feature extraction section has three blocks. Each block consists of a convolutional layer, batch normalization layer, rectified linear unit (ReLU), dropout layer and a max-pooling layer. The convolutional layer, which contains 6, 16, and 32 filters in respective blocks, generates feature maps by convolution operation across the previous layers’ input.

The classifier section contains the fully connected layer, softmax layer and the classification layer. The output from the fully connected layer goes to the softmax layer that computes the probabilities that the input image belongs to a particular class. Finally, the classification layer displays the most probable class.

To prevent overfitting and make the CNN model robust to variations in training data, we performed data augmentation by randomly rotating and translating the training data. The hyperparameters for training are mini-batch size 16, maximum epochs 100, and learning rate 0.0003. The network parameters were updated using stochastic gradient descent with a momentum optimizer

### Generation of 3D saliency map

**Fig. 2:**
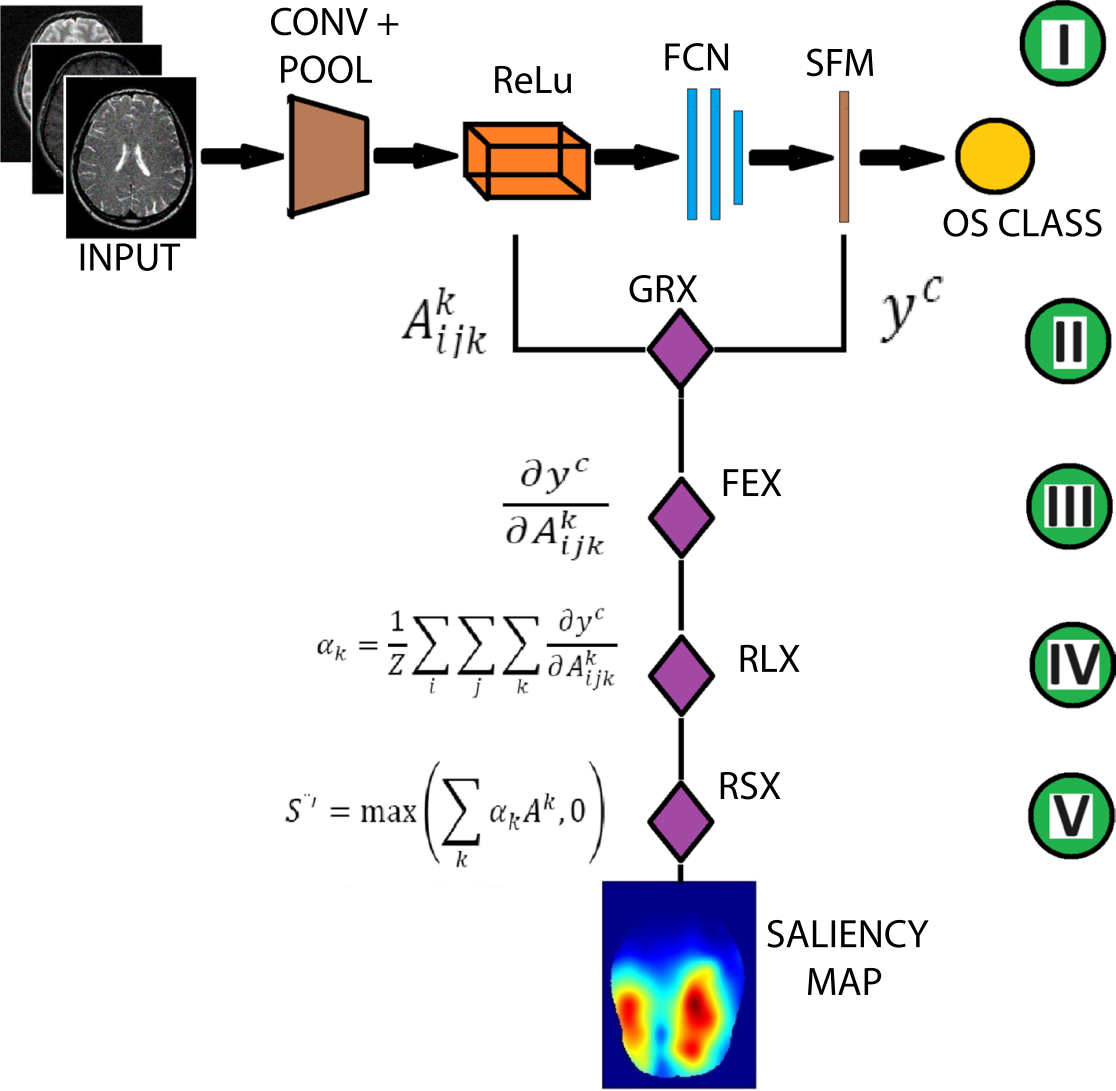
Five of the eight successive steps in the generation of a 3D saliency map. (I), A 3D MRI volume is fed to a trained CNN model, consisting of convolutional (CONV), pooling (POOL), rectifier linear unit (ReLU), fully connected (FCN) and softmax (SFM) layers, to predict an OS class. (II). Compute the gradient (GRX) of class scores extracted from the SFM with respect to the feature maps at the output of the ReLU. (III). The gradient-based feature map are spatially pooled (FEX) to obtain spatial importance of the feature maps. (IV). Application of ReLU function (RLX) on the spatially pooled feature maps to compute the cumulative spatial importance activations that contribute to the class discriminative localization map. (V). The resampling and alignment of the spatial importance activations to match the size and orientation, respectively, of the input image

A saliency map is a visualization method that provides insight on the operation of trained models. It reveals the contributions of pixels in the input image to the model prediction. In this study, the Grad-CAM architecture [14] developed for 2D images, was extended to 3D in eight successive steps (Fig. 2).

1. An MRI volume is fed into the trained model to predict its OS class *c*
2. Information is extracted from two layers of the trained model. The first layer is the softmax, where we extract the classification score $y^{c}$ corresponding to the predicted class c of the image. The second layer is the last convolutional layer, ReLU with *K* feature maps. We extract feature map $A^{k}_{ijk}$ with activations at location (*i*, *j*, *k*) of the feature map $A^{k}$. Then, computation of the gradient $\frac{\partial y^{c}}{\partial A^{k}_{ijk}}$ of the classification score $y^{c}$ of the input image with respect to the feature map $A^{k}_{ijk}$ of the last convolutional layer.
3. The gradient-based feature maps are spatially pooled using global average pooling. This gives the spatial importance or weights $\alpha _k$ for each feature map:
  1 $$\begin{aligned} \alpha _k = \frac{1}{Z}\sum \limits _{i}\sum \limits _{j}\sum \limits _{k} \frac{\partial y^{c}}{\partial A^{k}_{ijk}} \end{aligned}$$
  where *Z* is a normalization constant.
4. A ReLU function is applied to compute the cumulative spatial importance activations $H^{'}$ that contribute to the class discriminative localization map:
  2 $$\begin{aligned} H^{'} =\max \{\Sigma _{k} (\alpha _{k} A^{k},0)\} \end{aligned}$$
5. The data undergoes processing, first by resampling, using linear interpolation, followed by alignment to match the size of $H^{'}$ to the size $240 \times 240 \times 90$ and orientation, respectively, of the input image $I_{train}$.
6. The processed data is averaged to obtain the saliency map, $H_S$, $H_M$ and $H_L$, for short, medium and long survivors, respectively.
7. Slices in the $240 \times 240 \times 90$ average saliency map are matched to corresponding slices in $240 \times 240 \times 155$ SRI 24 brain template
8. Finally, we compute masks corresponding to foreground regions of slices in the SRI 24 template and then apply these masks to eliminate outliers outside the silhouette in corresponding slices of the newly indexed saliency maps.

### Probability maps

The saliency maps $(H_S,H_M,H_L)$ for each OS class were converted to corresponding probability maps $(D_S,D_M,D_L)$ that describe the probability of the event-of-interest at each pixel location such that:

3 $$\begin{aligned} D_{S} + D_{M} + D_{L}=1 \end{aligned}$$

The first step to compute the probability map is to rescale pixels in the saliency maps for each OS class to have a value between 0 and 1. Rescaling is necessary because the range of pixel values in the saliency map differs for each OS class. Thereafter, we compute the common weight w for each OS class.

4 $$\begin{aligned} w=\frac{I}{L_{H}} \end{aligned}$$

where I is a matrix of ones having the same size as the saliency maps and $L_H$ is the linear combination, with equal weights, of the rescaled saliency map.

5 $$\begin{aligned} L_{H}= H_{S} + H_{M} + H_{L} \end{aligned}$$

The probability map $(D_S,D_M,D_L)$ for each OS class is obtained by multiplying each saliency map with the common weight.

6 $$\begin{aligned} D_{S}= wH_{S} \end{aligned}$$

7 $$\begin{aligned} D_{M}= wH_{M} \end{aligned}$$

8 $$\begin{aligned} D_{L}= wH_{L} \end{aligned}$$

### Brain regions associated with survival classes

**Fig. 3:**
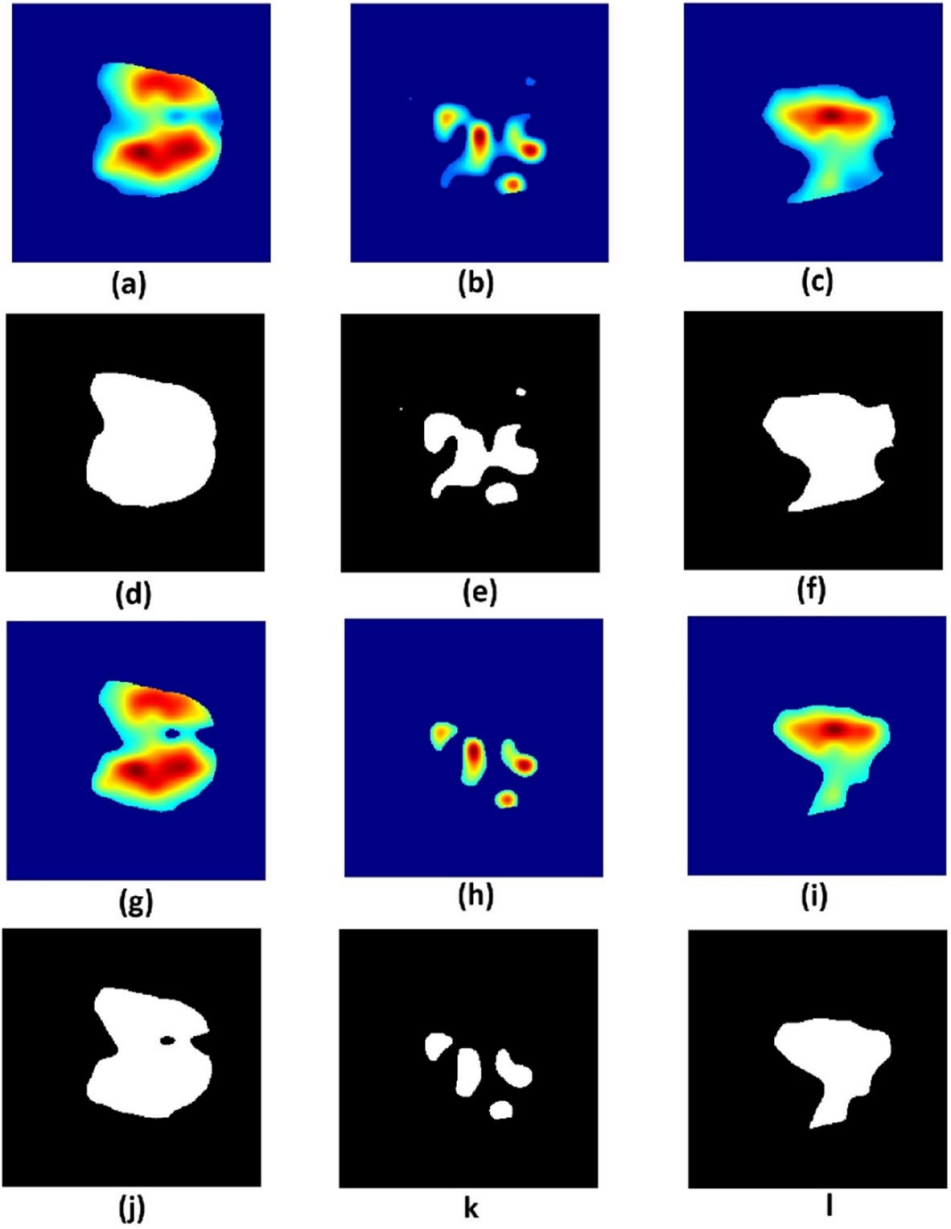
(First row) A slice in (**a**) short-term, (**b**) medium-term and (**c**) long-term survival saliency maps at threshold value 0.2 and (second row) their corresponding binary images. (Third row) A slice in (**g**) short-term, (**h**) medium-term and (**i**) long-term survival saliency maps at threshold value 0.3 and (fourth row) their corresponding binary images

The combination of 3D saliency map and SRI 24 brain atlas allows us to identify brain regions that are associated with each OS class. In order to demonstrate the variations in clinically relevant image regions that contributed to the model, we set global thresholds at $th=\{0.1,0.2,0.3,0.4,0.5,0.6\}$ on the saliency map to generate corresponding binary maps $(H^{0.1}, H^{0.2}, H^{0.3}, H^{0.4}, H^{0.5}, H^{0.6})$ for that threshold level. Figure 3 shows example binary images of a selected slice number of the saliency map for each OS class for threshold values of 0.2 (Fig. 3d-f) and 0.3 (Fig. 3j-l). Successive threshold values of 0.2 and 0.3 were selected as example images to demonstrate how gradual changes in tumor size and extent impacted the model outcome. Thereafter, we multiply a binary version of *H* with the SRI 24 brain atlas. This gives a product image $(\hat{H}^{th}_{S},\hat{H}^{th}_{M},\hat{H}^{th}_{L})$ for each OS class at threshold value *th*. Finally, we apply set operations [24] on the product images to identify tumor locations $\textbf{B}$ that are common to all the OS classes:

9 $$\begin{aligned} \textbf{B}= \bar{H}^{th}_{S} \cap \left(\bar{H}^{th}_{M} \cap \bar{H}^{th}_{L}\right) \end{aligned}$$

and the tumor locations $\textbf{B}_{S}, \textbf{B}_{M}, \textbf{B}_{L}$ that are uniquely associated to each OS class:

10 $$\begin{aligned} \textbf{B}_{S}= \left(\bar{H}^{th}_{S} - \bar{H}^{th}_{M} - \bar{H}^{th}_{L}\right) \end{aligned}$$

11 $$\begin{aligned} \textbf{B}_{M}= \left(\bar{H}^{th}_{M} - \bar{H}^{th}_{L} - \bar{H}^{th}_{S}\right) \end{aligned}$$

12 $$\begin{aligned} \textbf{B}_{L}= \left(\bar{H}^{th}_{L} - \bar{H}^{th}_{S} - \bar{H}^{th}_{M}\right) \end{aligned}$$

### Prediction of overall survival

**Fig. 4:**
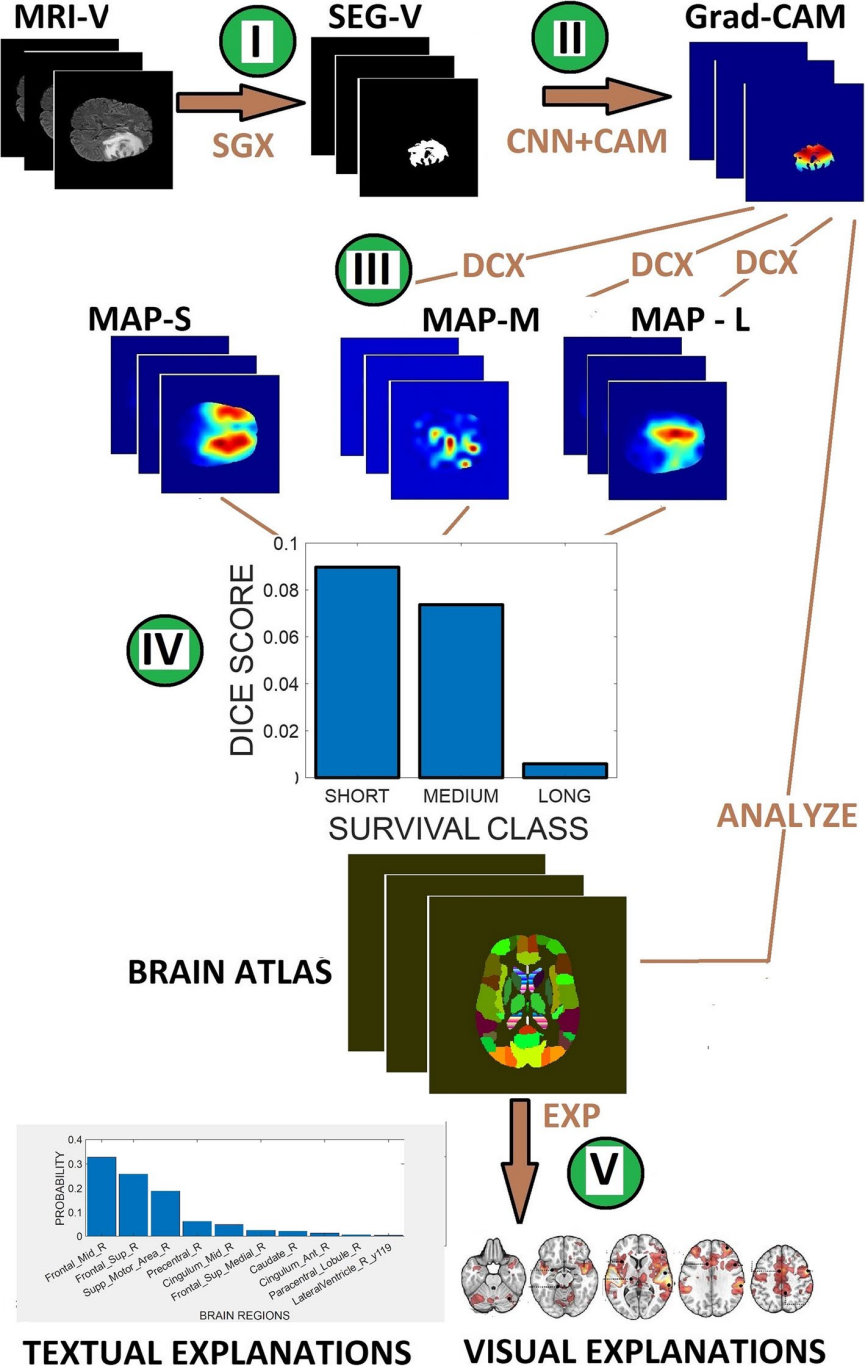
Flow chart for enhancing the interpretability of deep learning model in the prediction of glioma patient OS class. (I). The patient MRI volume (MRI-V) is segmented (SGX) to extract tumor regions (SEG-V). (II). The segmentation mask passes through a CNN (trained for OS classification) and fitted with Grad-CAM to extract saliency map. (III). Thereafter, the computation ((DCX) of the overlap, expressed by the dice score, between the saliency map and the probability map (MAP-S, MAP-M, MAP-L) representing each OS class. This step measures the probability of the event-of-interest. (IV) The predicted OS class is the OS class with the maximum dice score. (V) Application of histogram distribution and set theory (ANALYZE) on the saliency map and SRI 24 brain atlas provides visual and textual information that allows the physician to understand how and why the model makes predictions

Aside from the trained model, our proposed method predicts patient overall survival from the analysis of pixel-wise similarity matching between the patient multi-spectral MRI volumes, deep learning-derived 3D saliency map, and a brain-region atlas. Furthermore, the proposed method enhances the interpretability of a deep learning model (see Fig. 4).

1. The prediction begins with the segmentation of the patient’s MRI volume to extract tumor region.
2. The segmented MRI volume is fed to a CNN model trained for the prediction of OS class.
3. Application of Grad-CAM in conjunction with the trained model generates 3D feature map. The algorithm displays the feature map with range of colors depicting feature relevance (dark red color indicating highest relevance), thus allowing the physician to visualize the contribution of the different tumor regions to the prediction of the patient OS class.. Rescale the Grad-CAM-derived feature map to the same pixel intensity scale as the probability map $D_{c}$ for each OS class *c*.
4. Compute the Dice similarity score *Dice* between the patient Grad-CAM-derived feature map and the probability map $D_{c}$ for each OS class. The probability of the event-of-interest for the patient with respect to each OS class is
  13 $$\begin{aligned} Pr(Event)_{short}= Dice_{short} \end{aligned}$$
  14 $$\begin{aligned} Pr(Event)_{medium}= Dice_{medium} \end{aligned}$$
  15 $$\begin{aligned} Pr(Event)_{long}= Dice_{long} \end{aligned}$$
5. The predicted OS class of the patient $P_{OS}$ is the class with the maximum Dice score.
  16 $$\begin{aligned} P_{OS}= \max \{Pr(Event)_{short}, Pr(Event)_{medium}, Pr(Event)_{long}\} \end{aligned}$$
6. Convert the Grad-CAM-derived feature in step 3 to a binary image by setting a specific global threshold value.
7. Multiply the threshold-derived binary image in step 6 with SRI 24 brain atlas. Analysis of the nonzero pixels of the product images provides the physician with visual and textual explanations of the brain regions and their degrees of contributions to the predicted patient OS class.

### Statistical analysis

The indices of pixels in the brain atlas are analyzed using histogram to determine brain regions associated with each OS class. The contributions of brain regions to a patient survival were quantified by the dice score between tumorous MRI volume and different thresholds of saliency maps. The spearman rank correlation and the group scatter plot quantifies the overlap between brain MRI volume and different thresholds of the saliency map for each OS class. The overlap provide information on the brain regions associated with survival.

## Results

The proposed method was evaluated based on classification accuracy; the ratio of the number of correct predictions to the total number of predictions made. Table 2 display the classification accuracy of our proposed method with various threshold levels for the classification of OS. The best OS prediction accuracy of 0.552 was recorded for saliency map with $th=0.6$. This performance is comparable to the top 10 teams at the BraTs 2020 challenge [22] .

**Table 2:** Prediction accuracy of a patient OS class based on the number of correctly predicted patients’ survival with respect to the three classes of OS and according to different thresholds of the saliency map

Saliency Map Threshold Values	Overall Survival Classification Accuracy
0.0	0.517
0.1	0.517
0.2	0.517
0.3	0.483
0.4	0.448
0.5	0.483
0.6	0.552

**Fig. 5:**
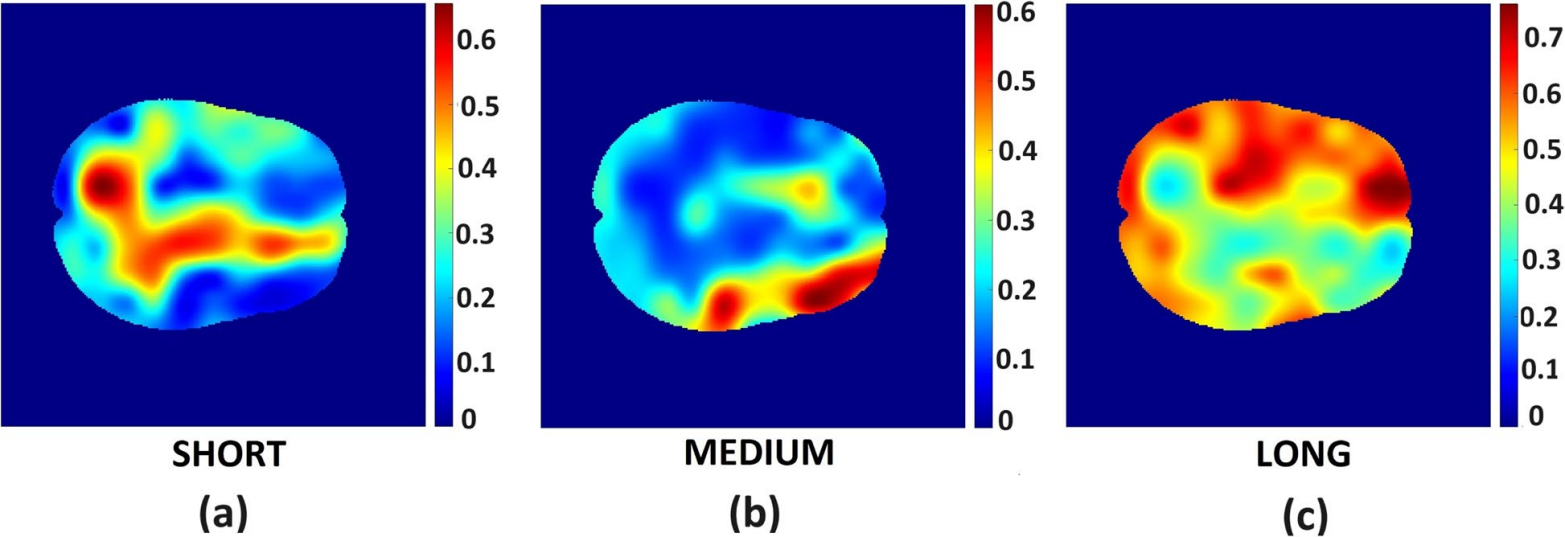
A slice in the saliency map for (left) short-term, (middle) medium-term and (right) long-term survival classes showing contributions of midbrain regions to the prediction of OS class. The levels of contributions vary from blue (least significant) and dark red (most significant) contribution

**Fig. 6:**
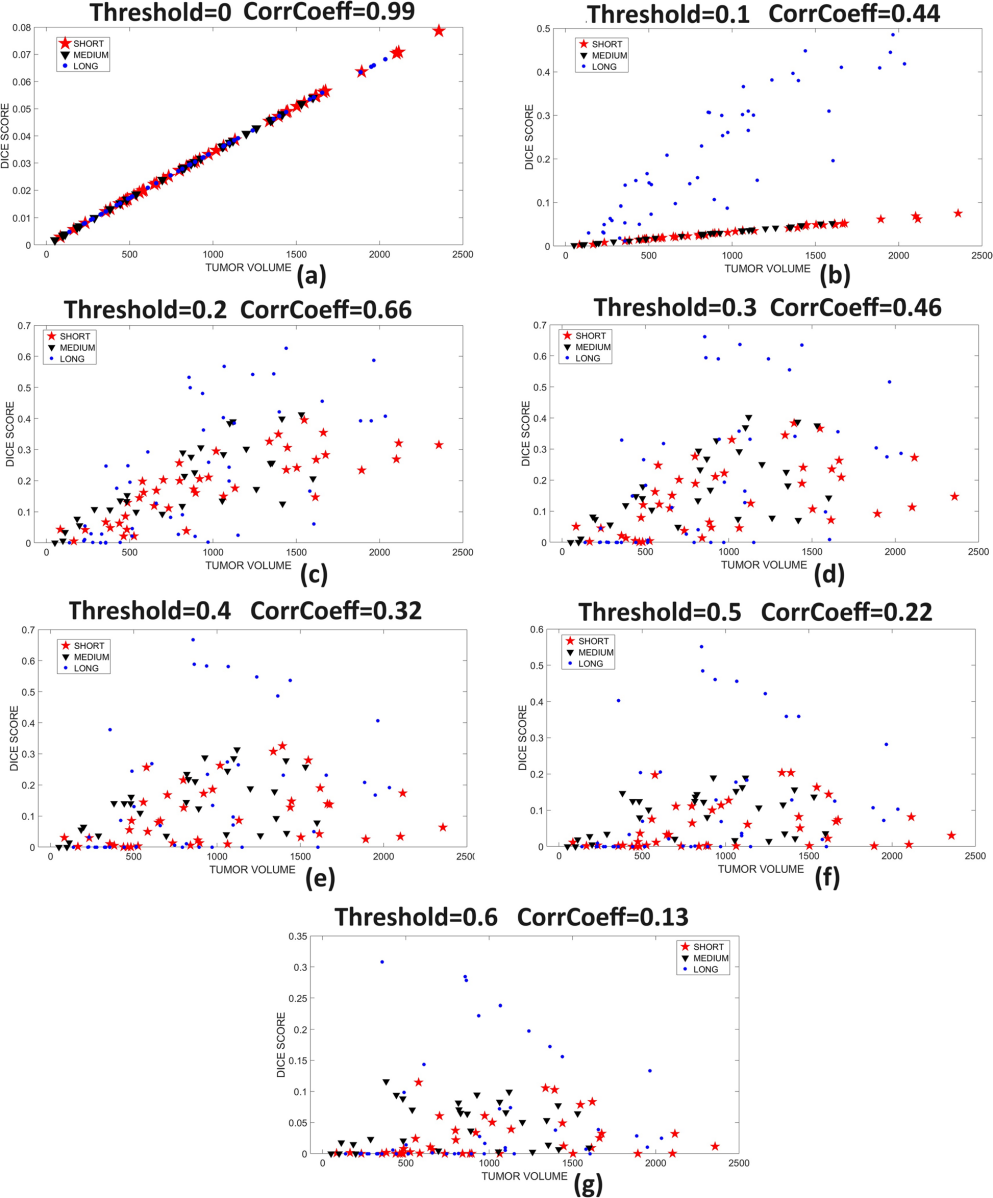
The overlap between tumor region and the saliency map at different threshold values. Plot of tumor volume and the dice score between tumor volume and saliency map, grouped by short-term, medium-term, and long-term survival at saliency map threshold values (**a**) 0, (**b**) 0.1, (**c**) 0.2, (**d**) 0.3, (**e**) 0.4, (**f**) 0.5 and (**g**) 0.6. The lower threshold values such as (a) and (b) with more tumor volumes show stronger correlation with saliency map

Example data of the saliency map for the short, medium, and long survival classes are displayed in Fig. 5a-c. Brain regions that are unique to short-term, medium-term, and long-term survival are displayed in Table 3. The spearman rank correlation coefficient between the overlap of tumor volume of each OS class and different thresholds of saliency map (based on Dice score) and relative tumor volumes are presented in Table 4. Relative volume is the ratio of the tumor volume for each OS class to the total MRI volume. Figure 6a-f depict the scatter plot of training images tumor volume and dice score grouped by short-term, medium-term, and long-term survivors. The scatter plot in Fig. 6a is a control, with no threshold (all pixels included), Figure 6a-b shows that there is maximum and strong correlation between tumor volumes and the saliency map, respectively. The high correlation is attributed to larger tumor volume associated with lower threshold values of 0 and 0.1, respectively. In Fig. 6c-f, the strength of correlation increases with more tumor overlap with specific brain region for each OS class.

**Table 3:** Brain regions that are unique to short-term, medium-term and long-term survival are displayed in the first, second and third columns, respectively

Short-term Survival	Medium-term Survival	Long-term Survival
Olfactory_L	Frontal_Mid_L	Precentral_R
Insula_L	Frontal_Inf_Tri_L	Frontal_Sup_L
Hippocampus_L	Frontal_Inf_Orb_L	Frontal_Sup_R
ParaHippocampal_L	Temporal_Mid_L	Frontal_Mid_R
Cuneus_R	Temporal_Mid_R	Frontal_Inf_Oper_R
Precuneus_L	Temporal_Pole_Mid_R	Frontal_Inf_Tri_R
Putamen_L	Temporal_Inf_L	Rolandic_Oper_R
Pallidum_L		Supp_Motor_Area_L
Thalamus		Supp_Motor_Area_R
LateralVentricle_L_y107-113		Frontal_Sup_Medial_R
		Insula_R
		Cingulum_Ant_R
		Cingulum_Mid_L
		Cingulum_Mid_R
		Postcentral_R
		Parietal_Sup_R
		Parietal_Inf_R
		SupraMarginal_R
		Paracentral_Lobule_R
		Caudate_R
		Putamen_R
		LateralVentricle_R_y113-148
		CorpusCallosum_AP_0
		CorpusCallosum_AP_1
		CorpusCallosum_AP_2
		CorpusCallosum_AP_3
		CorpusCallosum_AP_4

**Table 4:** Spearman rank correlation between the overlap of tumor volume of each OS class and different thresholds of corresponding saliency map (based on Dice score) and relative tumor volume

Saliency Map Threshold Value	Short-term Survival	Medium-term Survival	Long-term Survival
0.0	0.9999	1.0000	1.0000
0.1	0.9966	0.9993	0.8797
0.2	0.7995	0.7544	0.6500
0.3	0.4891	0.6002	0.4924
0.4	0.3199	0.4666	0.3823
0.5	0.2720	0.3236	0.2748
0.6	0.2601	0.1359	0.1529

## Discussion

The probability of survival, derived from studies on a population-based cohort of glioma patients, is the basis for describing information on a patient survival after glioma diagnosis [25]. Our study proposes converting average saliency maps from patients in three distinct OS classes to a new probability map that provides information on how important various image pixels and brain regions are for determining OS using a deep learning model.

The anatomical atlas is an important tool used in brain image analysis for the identification of tumor locations. Our study combines information from binary maps of training images’ saliency map for each OS class with the SRI 24 brain atlas. Our results suggest that a patient overall survival is dependent on tumor location in specific brain regions. Tumors located in the hippocampus, thalamus, left insula and regions of the left lateral ventricle are associated with short survival. Tumors located in frontal and temporal lobes are associated with medium survival while tumors located in corpus callosum, right insula and the regions of the right lateral ventricle are associated with long survival. Our findings are consistent with several studies, which find relationship between overall survival and tumor location. Fyllingen et al. [26] suggest that central tumor location was associated with short survival while survival is favorable according to the distance between the center of the third ventricle and the contrast-enhancing tumor border. Another study [27] found that patients with tumors in non-eloquent areas of the brain have favorable survival compared to patients with tumors in eloquent or near-eloquent areas regardless of resection extent. In Roux et al. [28], the study suggests that a glioblastoma patient survival is affected by the subventricular zone involvement and differences in tumor location.

Our study also suggests that an enlarged tumor volume expressed by its size and extent is associated with shorter survival within each OS class. This finding is consistent with previous work [29] where data from multicenter and single-center trials show that pre-treatment contrast-enhanced tumor volume is a strong prognostic factor in determining OS in a cohort of patients with recurrent glioblastoma. One of the findings in Ellingson et al. [29] is shorter survival in patients with large tumors. In this study, there was significant survival advantage in patients with enhancing tumor volume less than 2*cm* in diameter compared with patients with larger tumor volume $>2cm$.

Our study has limitations. First, since our dataset is retrospective, we do not have control over the composition of the study population and data size. The study population consisting of 30 short, 42 medium and 46 long survivors is imbalance with the risk of a study outcome that is prone to bias. Our deep learning approach will arguably work more efficiently with a large datasets to reduce the risk of overfitting. Second, the study utilizes both low- and high-grade glioma, and information on the relative percentage of the two grade types was not provided in the BraTs 2020 challenge. Third, we do not have the full clinical information on the patients, including positioning of the radiotherapy dose distribution as well as any salvage chemotherapy that may influence patient outcome. Finally, the influence of image features was based on dice-score derived variables rather than volumetric variables.

## Conclusions

In this study, we propose to enhance the interpretability of a deep learning model for glioma patient survival predictions by including saliency-based maps that represent features unique for the survival classes. This explainable AI may help show the impact of the tumor location on patient survival, including how a tumor association with eloquent brain regions return shorter survival. This information can assist physicians in understanding how deep-learning models make its predictions.

## Data Availability

We used BRATS2020 dataset for training and validation of model. Brats2020 is available with permission from https://www.med.upenn.edu/cbica/brats2020/data.html.

## Abbreviations

MRI: Magnetic Resonance Imaging
Grad-CAM: Gradient Weighted Class Activation Mapping
CNN: Convolutional Neural Network
OS: Overall Survival
IDH: Isocitrate dehydrogenase
MGMT: O6-methylguanine-DNA-methyltransferase
XAI: Explainable artificial intelligence
SHAP: Shapley additive explanations
T1: T1-weighted MRI
T1c: T1-weighted post-contrast MR1
T2: T2-weighted MRI
FLAIR: Fluid-attenuated inversion recovery
ReLU: Rectified linear unit
